# GERO-EDUCATION IN JAPAN: COMMUNITY RE-DESIGNING TO ACHIEVE HEALTHY AGING AND WELL-BEING

**DOI:** 10.1093/geroni/igad104.1091

**Published:** 2023-12-21

**Authors:** Katsuya Iijima

**Affiliations:** The University of Tokyo, Tokyo, Tokyo, Japan

## Abstract

Japan has achieved the world’s highest level of life expectancy and is currently experiencing a super-aging society. Because of this, we have to take steps towards achieving a healthy aging society with evidence-based policies and longevity with happiness (well-being). In addition, it is necessary to integrate digitalization with information and communications technology through more than just the conventional healthcare measures such as going into the local community to increase opportunities for people from all generations to connect and interact. We also have to realize a true “population approach” with the aim of building a community where residents can easily engage in community-based activities centered on self-help and mutual aid. It is an urgent task to create a place for activities such as lifelong education and multi-generational exchanges as well as to build a comprehensively integrated community care system that allows people to live with peace of mind when they need long-term care. Thinking about planning the 100-year life, the super-aging society has had consequences for the healthcare and social care of older people in Japan, leading to societal innovations that promote health in older people. To extend the healthy life expectancy and achieve a bottom-up sense of community resilience and activity, an interdisciplinary gerontological collaboration with multi-stakeholders is indispensable, with collaborators such as municipal governments, industries, professional staffs, academic researchers (including universities, young career researchers, and students), and citizens. This session will introduce our efforts to promote gerontology education through redesigning the community with comprehensive knowledge.

